# Influence of Cross-Regional Cultivation on the Flavor Characteristics of *Pyropia haitanensis*

**DOI:** 10.3390/foods15010181

**Published:** 2026-01-05

**Authors:** Yuting Zhang, Qijun Luo, Juanjuan Chen, Rui Yang, Wenrong Zhu, Haimin Chen

**Affiliations:** 1 Collaborative Innovation Center for Zhejiang Marine High-Efficiency and Healthy Aquaculture, Ningbo University, Ningbo 315211, China; 2011042034@nbu.edu.cn (Y.Z.); luoqijun@nbu.edu.cn (Q.L.); chenjuanjuan@nbu.edu.cn (J.C.); yangrui1@nbu.edu.cn (R.Y.); 2 Xiangshan Xuwen Seaweed Development Company, Ningbo 315211, China; seaweedzhu@139.com

**Keywords:** *Pyropia haitanensis*, cross-regional cultivation, odor-active compounds, umami taste, environmental factors

## Abstract

Geographical relocation can alter flavor quality in marine crops. Here, the same cultivar of *Pyropia haitanensis* (“ZHEDONG 1”) was cultivated at six sites spanning northern to southern China, and taste- and odor-active compounds were characterized by LC–MS and GC×GC–TOFMS together with environmental measurements. Inosine monophosphate was identified as the major contributor to umami intensity and showed a strong positive association with nitrate levels. A conserved core aroma profile dominated by heptanal, 2-pentylfuran, nonanal, and 2-ethyl-1-hexanol was consistent across regions, whereas differences in their relative abundances led to distinct regional sensory nuances. Correlation analyses further indicated that phosphate, temperature, and pH shaped volatile composition. These results demonstrate that while *P. haitanensis* retains a genetically determined intrinsic flavor, environmental conditions modulate flavor intensity and aromatic complexity during cross-regional cultivation.

## 1. Introduction

Flavor is a critical quality attribute of agricultural products, directly shaping consumer preference and market value. The sensory attributes of agricultural commodities are not solely determined by genetic background but are also profoundly influenced by environmental factors [1]. Products of the same variety often display distinct sensory characteristics when cultivated under different environmental conditions. This phenomenon has been extensively documented in terrestrial plants. For example, a multi-region study on fine Australian wines showed that the same Chardonnay and Shiraz varieties exhibit regionally distinctive sensory and volatile signatures attributable to terroir effects (soil, microclimate, water) [2,3]. In rice, regional differences (Xiangshui vs. Hangzhou) in amylose, protein, and amino acids significantly affected eating quality, with Xiangshui samples showing higher sensory scores [4]. Similarly, Mikrou et al. showed that monovarietal extra olive oils from different Greek regions (Crete, Peloponnese, Lesvos) exhibited distinct volatile profiles, with specific compounds serving as reliable markers of geographic origin [5]. These examples highlight that geographic relocation or environmental heterogeneity can significantly reshape product flavor.

In marine agriculture, economically important seaweeds are no exception. They are extensively cultivated across East Asia, with *Porphyra* sensu lato representing one of the most valuable groups of red algae, contributing over 95% of global laver production [6]. Recent studies have confirmed that the quality of *Porphyra* is dynamically shaped by environmental conditions. For example, *Neopyropia yezoensis* produced in northern sites of China has been reported to exhibit lower product quality (e.g., gloss, texture) than traditional Jiangsu products, highlighting strong environmental effects on edible quality [7]. Likewise, *N. yezoensis* cultivated across different sites in Korea showed significant variability in amino acid, and mineral content [8].

Laver products are appreciated not only for their nutritional benefits, but also for their characteristic umami and aroma [9]. The flavor of seaweed arises from a complex interaction of soluble taste-active compounds, primarily free amino acids (FAAs) and 5′-nucleotides, and volatile organic compounds such as aldehydes, alcohols, ketones, and esters [10,11,12]. Key taste-active substances include glutamic acid (Glu), aspartic acid (Asp), and inosine monophosphate (IMP), which confer umami, as well as alanine (Ala), glycine (Gly), and serine (Ser), which impart sweetness [13]. Aroma-active volatiles such as nonanal, hexanal, and benzaldehyde provide additional layers of sensory complexity [14,15]. Recent research has shown that the composition of these flavor substances is not constant but varies according to environmental conditions such as nutrient availability, salinity, and light intensity [16]. Miyasaki et al. reported a strong association between the quality of laver and the accumulation of volatile compounds [17]. Likewise, nitrate enrichment has been linked to elevated levels of total protein and umami-related FAAs, thereby enhancing the umami taste of *Pyropia haitanensis* [18]. These findings parallel the well-established crop science evidence that local growth conditions significantly shape product flavor profiles.

*P. haitanensis* is the principal seaweed cultivated in China. Historically, its cultivation was concentrated in Zhejiang and Fujian provinces, but in recent decades, its cultivation area has expanded northward to Jiangsu, Shandong, and even Liaoning, driven by climate change and industry demand [18]. Although this geographic expansion has increased production scale and economic value, it has also raised concerns regarding product quality consistency. Farmers and processors have noticed that laver grown outside its traditional range sometimes exhibits altered texture, which may influence market price.

Against this backdrop, it is essential to evaluate how cultivation environments shape the flavor quality of *P. haitanensis*. Although previous work has described general correlations between seaweed chemistry and environment, comprehensive multi-regional analyses using standardized strains and metabolomic approaches remain limited. To address this gap, the present study investigates flavor differences in a single cultivar of *P. haitanensis* cultivated across six major seaweed farming regions in China. By integrating LC–MS/MS and GC×GC–TOFMS with measurements of environmental parameters, we aim to identify the key taste- and odor-active compounds, quantify their regional variability, and assess their relationships with seawater nutrient regimes and physicochemical conditions. This study seeks to elucidate the ecological drivers of flavor formation in *P. haitanensis*, thereby providing a scientific basis for optimizing site selection, and maintaining product quality, supporting the sustainable development of the laver industry under changing climatic and geographic conditions.

## 2. Materials and Methods

### 2.1. Seaweed Culture and Sample Collection

**Figure 1:**
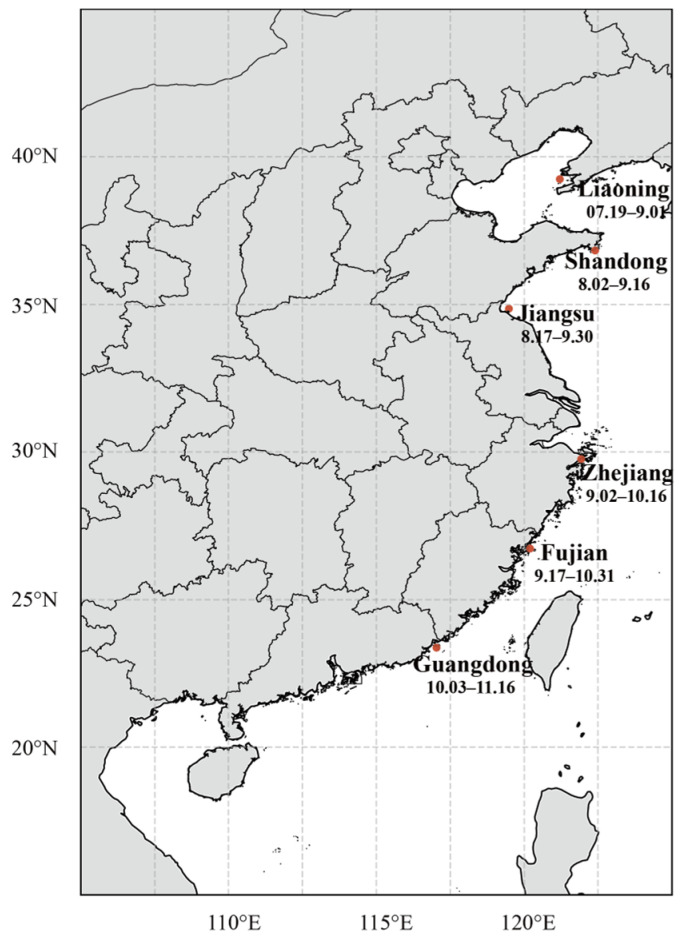
Sampling sites for *Pyropia haitanensis* cultivation along the Chinese coastline. The dates denote the cultivation period from seeding-net deployment to harvest at each location.

In 2023, the same strain of *P. haitanensis* “ZHEDONG 1” (GS-01-013-2014), obtained from the Algal Germplasm Resource Bank of Ningbo University, was cultivated at six coastal sites spanning the Chinese coastline from north to south (Figure 1). The sites included Dalian, Liaoning Province (39°18′17″ N; 121°22′54″ E); Weihai, Shandong Province (36°49′7″ N; 122°13′47″ E); Lianyungang, Jiangsu Province (34°47′49″ N; 119°25′39″ E); Ningbo, Zhejiang Province (29°42′47″ N; 121°54′13″ E); Zhangzhou, Fujian Province (23°57′52″ N; 117°47′0″ E); and Shantou, Guangdong Province (23°24′49″ N; 117°2′18″ E). These six locations represent the principal farming areas for *P. haitanensis* within their respective provinces.

The free conchocelis of the “ZHEDONG 1” strain was inoculated onto shells and cultivated in local farmers’ nurseries until the conchosporangia matured. According to the seawater temperature at each site, the matured conchosporangia were transported to the respective cultivation areas, where they released conchospores that attached to cultivation nets. These seeded nets were subsequently deployed in the sea for grow-out, where nets floated on the sea surface to ensure a consistent cultivation depth. No artificial fertilizers were applied, and the seaweed relied entirely on natural seawater nutrients and tidal flow for growth and water exchange. After 45 days of cultivation, at the time of the first harvest, samples were collected from each site.

At each cultivation site, three nets were randomly selected as biological replicates. From each net, blades were sampled from four peripheral and two central positions, and subsequently pooled to form one composite sample. A portion of the cleaned thalli was placed in a refrigerated container for phenotypic trait assessment, while the remaining material was immediately snap-frozen in liquid nitrogen. In parallel, 1 L of surface seawater was collected at each site, divided into two sterile 500 mL bottles, stored at 4 °C in the dark, and transported to the laboratory. In the laboratory, frozen algal tissues were ground under liquid nitrogen for metabolite analysis. For fresh samples, blade length, width, and fresh weight were measured (*n* = 15).

### 2.2. Detection of Free Amino Acids

Freeze-dried samples (50 mg, *n* = 3), previously ground in liquid nitrogen, were extracted with 10 mL of 75% ethanol by boiling for 30 min. After centrifugation at 3000× *g* for 10 min, the supernatant was collected, while the pellet was resuspended in 2 mL deionized water and sonicated at 80 °C for 20 min. The resulting extracts were combined, centrifuged again, and the pooled supernatants were evaporated to dryness, then re-dissolved in 1 mL deionized water for analysis by LC–MS. Instrument stability was monitored using pooled Quality Control (QC) samples injected after every 10 experimental samples.

Liquid chromatography was performed on an Acquity I-Class UPLC system (Waters Corp., Milford, MA, USA) coupled with a Xevo TQ-S Micro tandem quadrupole mass spectrometer. Separation was achieved on an ACQUITY UPLC^®^ HSS T3 column (1.8 µm, 2.1 × 150 mm) maintained at 35 °C, with a flow rate of 0.35 mL min^−1^. The mobile phase consisted of 10 mmol L^−1^ ammonium acetate containing 0.1% formic acid (solvent A) and methanol with 0.1% formic acid (solvent B). The gradient program was as follows: 0–0.6 min, 98% A; 0.6–0.8 min, 98–80% A; 0.8–3.0 min, 80–90% A; 3.0–5.0 min, 90–98% A.

Mass spectrometry was carried out in positive electrospray ionization mode, with a capillary voltage of 4.0 kV, cone voltage of 30 V, and collision energy of 33 eV. Nitrogen was used as the desolvation gas at 550 °C and 650 L h^−1^. Data were collected using MassLynx v4.1 software (Waters Corp.). FAAs were detected in multiple reaction monitoring mode following the method of Yuan et al. [13]. Quantification was performed by comparing sample retention times and mass spectra with those of authentic amino acid standards (Sigma-Aldrich, Saint Louis, MO, USA), and calibration curves were used to calculate concentrations.

### 2.3. Detection of Nucleotides

Freeze-dried material (0.2 g, *n* = 3) was subjected to ultrasonic extraction for 10 min with 5 mL of 5% methanol containing 0.05 mol L^−1^ phosphoric acid. The extracts were centrifuged at 3000× *g* for 10 min, and the resulting supernatants were passed through 0.22 µm filters prior to analysis.

Nucleotides were quantified using an HPLC system (Waters 2695, Waters Corp., Milford, MA, USA) equipped with a Diamonsil C18 column (4.6 × 250 mm), maintained at 30 °C. The flow rate was set at 0.8 mL min^−1^. The mobile phases consisted of 5% methanol in water with 0.05 mol L^−1^ phosphoric acid (solvent A) and 80% methanol in water with 0.05 mol L^−1^ phosphoric acid (solvent B). The gradient program was as follows: 0–5 min, 100% A; 5–15 min, 100–90% A; 15–25 min, 90–0% A; 25–30 min, 0% A; 30–40 min, 0–100% A; and 40–45 min, 100% A. Detection was carried out at 254 nm using a photodiode array detector. Commercial nucleotide standards (Sigma-Aldrich) were analyzed in parallel to generate calibration curves, which were used for concentration determination.

### 2.4. GC×GC–TOFMS Analysis

Volatile compounds were enriched using headspace solid-phase microextraction (HS-SPME). Briefly, 0.5 g of freeze-dried seaweed powder was accurately weighed into a 20 mL headspace vial. Strict consistency in sample mass and headspace conditions was maintained to ensure the comparability of semi-quantitative data. A 1 cm 50/30 μm DVB/CAR/PDMS fiber (Supelco, Bellefonte, PA, USA) was used for volatile enrichment. The SPME procedure was performed automatically using a Gerstel MPS autosampler (Gerstel, Mülheim an der Ruhr, Germany). Prior to the first extraction, the fiber was preconditioned at 270 °C for 3 min to remove potential contaminants. For analysis, the sample vial was first equilibrated in a 40 °C water bath for 10 min. Subsequently, the fiber was exposed to the vial headspace for adsorption for 30 min at 40 °C. Finally, the fiber was introduced into the GC injector for thermal desorption at 250 °C for 5 min in splitless mode for online analysis.

Volatile profiling was performed using a LECO Pegasus HRT 4D Plus comprehensive two-dimensional gas chromatography time-of-flight mass spectrometry (GC×GC–TOFMS, LECO Corp., St. Joseph, MI, USA). Separation was achieved on a DB-Wax polar capillary column (30 m × 0.25 mm × 0.25 μm, Agilent Technologies, Santa Clara, CA, USA) with high-purity helium (≥99.999%) as the carrier gas. The injector temperature was maintained at 250 °C in splitless mode. The oven program was as follows: initial temperature 40 °C (held for 3 min), ramped to 230 °C at 10 °C/min, and held for 6 min. Carrier gas flow was set to 1.0 mL/min in constant-flow mode. Mass spectrometry conditions were as follows: ionization by electron impact (EI^+^) at 70 eV with an emission current of 1 mA; ion source and transfer line temperatures were set to 200 °C and 250 °C, respectively. The detector voltage was 2000 V. Data were acquired in full-scan mode across a mass range of 35–500 *m*/*z*. The acquired volatiles’ mass spectrum was annotated using LECO ChromaTOF software (version 5.51) by automated peak deconvolution and mass spectral matching against the NIST 17 database and ChromaTOF 4.3X database search software. To ensure robust identification, a rigorous dual-verification strategy was employed based on retention indices (RI) calculated from a homologous series of n-alkanes (C6–C26) analyzed under identical chromatographic conditions. For compounds with available literature retention data on polar columns, identification was considered valid only when the mass spectral match factor exceeded 700 and the difference between the calculated and literature RI was less than 20. In cases where specific polar library RIs were unavailable due to database limitations, a stricter match factor threshold greater than 750 was applied to maintain putative identification reliability.

Regarding quantification, a peak area normalization method was applied to determine the relative abundance of volatile compounds. Briefly, the peak area of each individual compound was integrated. To ensure data reliability, peaks detected in blank control sample (empty vial) and identified system artifacts were strictly excluded from the dataset. Subsequently, the relative content of each compound was calculated as a percentage of the total peak area of all retained volatiles in the sample (Area%).

### 2.5. Determination of Environmental Parameters

Before nutrient analysis, 400 mL of seawater was filtered through a 0.45 μm glass fiber membrane (GF/F, 50 mm, Whatman, Maidstone, UK) to remove suspended matter and phytoplankton. Dissolved inorganic nutrients, including ammonium (NH_4_^+^-N), nitrite (NO_2_^−^-N), nitrate (NO_3_^−^-N), and phosphate (PO_4_^3−^–P) were quantified using standard methods stipulated by GAQSIQ [19]. Temperature, salinity, and pH were recorded in situ using a HACH HQ4300 multiparameter meter (Hach Company, Loveland, CO, USA). The total sunshine duration during the cultivation period at each site was calculated based on daily sunshine records obtained from the China National Meteorological Information Center.

### 2.6. Assessment of Odor and Umami Potential

The relative odor activity value (ROAV) was used to assess the contribution of individual odorants to the overall aroma profile of the samples, and was calculated as follows [20]:

(1) ROAV=100×(CA÷TA)÷(Cmax÷Tmax)

where C represents the normalized concentration of a compound, T is its odor threshold, A denotes the compound of interest, and max refers to the compound with the highest ratio of concentration to threshold. Compounds with ROAVs ≥ 0.1 were defined as key aroma-active components.

Taste activity values (TAVs) were applied to evaluate the contribution of umami-related compounds to taste perception, and were calculated according to Gong et al. [21]:

(2) TAV=C1÷T

where C1 represents the concentration of a taste compound (mg/g), and T is the threshold concentration, i.e., the minimum level perceptible by human taste. A TAV greater than 1 suggests that the compound makes a notable contribution to the overall taste profile.

The contribution of FAAs and nucleotides to umami taste was evaluated by calculating the equivalent umami concentration (EUC) [21]. The EUC, expressed as grams of monosodium glutamate (MSG) per 100 g of sample, was determined using the following equation:

(3) $$EUC=\Sigma aibi+1218\left(\Sigma aibi\right)\times \left(\Sigma ajbj\right)$$

where EUC is grams of MSG per 100 g of sample. In this equation, 1218 is a synergistic constant. The variables ai and aj represent the quantity (g 100 g^−1^) of amino acids and nucleotides, respectively; bi represents the umami coefficients of amino acids to MSG (Glu = 1 and Asp = 0.077), and bj represents the umami coefficients of nucleotides to IMP (IMP = 1, guanosine 5′-monophosphate (GMP) = 2.3, and adenosine 5′-monophosphate (AMP) = 0.18).

### 2.7. Data Processing and Statistical Analysis

The sensory attributes of metabolites were identified using the Odor database and FlavorDB (https://cosylab.iiitd.edu.in/flavordb, accessed on 10 January 2025), which contains 25,595 flavor molecules covering a wide spectrum of taste and odor properties. FlavorDB was further applied to generate a network diagram of compound–flavor associations using the igraph package in R (version 4.3.1) [22]. Compound classification was carried out with the ClassyFire tool and PubChem (https://pubchem.ncbi.nlm.nih.gov/, accessed on 10 January 2025) [23], and the distribution and relative abundance of flavor-related metabolites in each class were assessed.

To explore overlaps across sites, an upset plot was produced with R to visualize shared volatiles and odor-active compounds (OACs) among the six groups. Principal component analysis (PCA) was performed using SIMCA-P (version 14.1, https://www.umetrics.com/, accessed on 5 March 2025) to describe data structure and identify discriminative variables. Prior to PCA, the data were processed using the mean centering (Ctr) scaling method to focus on the fluctuation of variations around the mean. Correlations between flavor/taste metabolites and environmental factors were examined by Mantel tests and Pearson’s correlation analysis. Incidence matrices were visualized with the pheatmap package in R (v3.3.2). The coefficient of variation (CV) was calculated as CV=SD÷Mean×100%.

Results for FAAs and nucleotides are expressed as mean ± SD. Differences among groups were evaluated by one-way ANOVA, followed by Duncan’s multiple range test or Tamhane’s T2 test for pairwise comparisons. Statistical analyses were performed with SPSS v27.0, and significance was accepted at *p* < 0.05.

## 3. Results

### 3.1. Growth Performance of P. haitanensis Across Cultivation Sites

**Figure 2:**
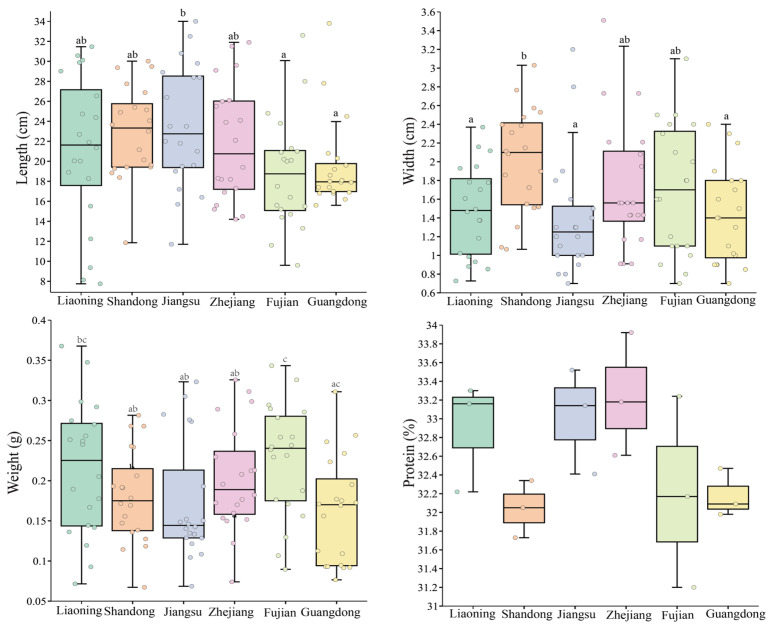
Production traits of *P. haitanensis* collected from different cultivation sites. Different superscript letters indicate significant differences (*p* < 0.05).

Regional differences were observed in blade morphology and protein content of *P. haitanensis* among the six cultivation sites (Figure 2). Blades from Jiangsu (23.51 ± 6.01 cm) and Shandong (22.89 ± 4.53 cm) were significantly longer than those from Fujian and Guangdong (*p* < 0.05). Shandong also showed the greatest blade width (2.02 ± 0.54 cm), while Jiangsu and Guangdong had narrower blades. Fresh weight also varied, with Fujian (0.23 ± 0.07 g) being significantly heavier than Guangdong (0.16 ± 0.07 g). In contrast, protein content remained relatively stable across sites, ranging narrowly between 32.04% and 33.24%. Zhejiang showed slightly higher values than others.

### 3.2. Regional Variation in Taste-Active Compounds and Umami Intensity of P. haitanensis

Differences in the composition of FAAs and taste-active nucleotides were observed among *P. haitanensis* cultivated in different regions, resulting in clear regional variation in taste characteristics (Table 1). Among umami amino acids, Glu showed consistently high levels across all samples, with TAVs generally exceeding 37, making it the primary source of umami. The highest Glu content was detected in Guangdong (18.84 ± 1.63 mg/g), which was significantly higher than that of other regions (*p* < 0.01), whereas Zhejiang exhibited the lowest (11.37 ± 1.11 mg/g). Asp ranged from 2.59 to 3.93 mg/g across sites, with TAVs above 1. For sweet-tasting amino acids, Ala exceeded 60 mg/g in Liaoning, Guangdong, and Jiangsu, with TAVs greater than 100, making it a key component enhancing sweetness. In addition, threonine (Thr) in Guangdong reached 5.18 mg/g (TAV 1.99), further reinforcing sweetness. In contrast, bitter amino acids such as leucine (Leu), phenylalanine (Phe), and isoleucine (Ile) were relatively high in some regions but showed low TAVs overall. However, lysine (Lys) levels were markedly elevated in Fujian and Guangdong (*p* < 0.01), with TAVs of 15.14 and 21.24, respectively.

Regarding taste-active nucleotides, IMP and GMP were the major contributors to umami. The Zhejiang samples contained the highest IMP level (205.37 mg/100 g, TAV 8.21, *p* < 0.01), indicating the strongest umami potential, whereas Guangdong exhibited relatively low IMP content, only slightly higher than that of Liaoning. In contrast, GMP levels in Zhejiang, Fujian and Guangdong were markedly higher than those in other regions (>25 mg/100 g, TAV > 2.0, *p* < 0.01). AMP showed little regional variation (TAV 0.2–0.37) and contributed only marginally to umami.

**Figure 3:**
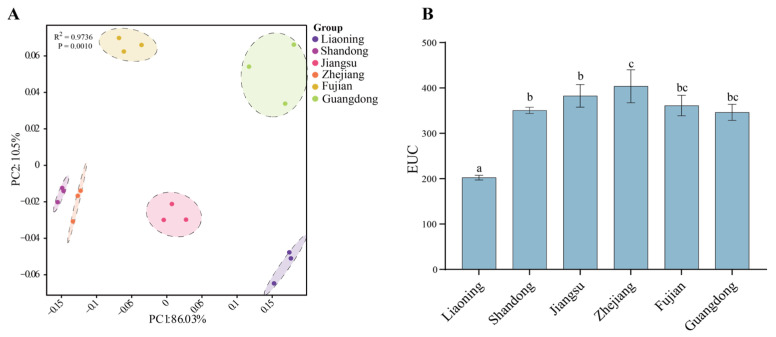
Taste profiles of fresh *P. haitanensis* cultivated in different cultivation sites. (**A**) Principal component analysis (PCA) of taste-related compounds from different provinces. (**B**) Equivalent umami concentrations (EUCs) in thalli collected from different sites. Different superscript letters indicate significant differences (*p* < 0.05).

The PCA results revealed clear separation among *P. haitanensis* cultivated in different regions based on their amino acid and nucleotide compositions (Figure 3A). According to the loading scores, PC1 was mainly driven by IMP and Ala. PC2 was largely influenced by GMP and Ala. Thus, IMP, GMP, and Ala emerged as the key taste-active compounds underlying regional differences in the taste of cultivated *P. haitanensis*. Samples from Guangdong and Liaoning were clearly separated from other regions, highlighting their distinct regional characteristics.

EUC analysis further highlighted regional differences in overall umami intensity among the samples (Figure 3B). The Zhejiang samples exhibited the highest EUC values, indicating the most pronounced umami taste, whereas the values declined progressively with increasing distance of transplantation to northern and southern regions. The lowest EUC was observed in Liaoning.

### 3.3. Regional Differences in Volatile Profiles and Key Odor-Active Contributors

The volatile compounds of *P. haitanensis* from different cultivation regions were analyzed, and clear regional variation in composition was observed (Table S1, Figures S1 and S2). In total, 1260 compounds were identified, with the number of volatiles ranging from 365 to 473 across sites. The Zhejiang samples contained the most compounds, whereas Liaoning had the fewest. Only 88 compounds were common to all regions, representing the core components. Shandong exhibited the largest number of region-specific compounds (142), followed by Zhejiang (141).

**Figure 4:**
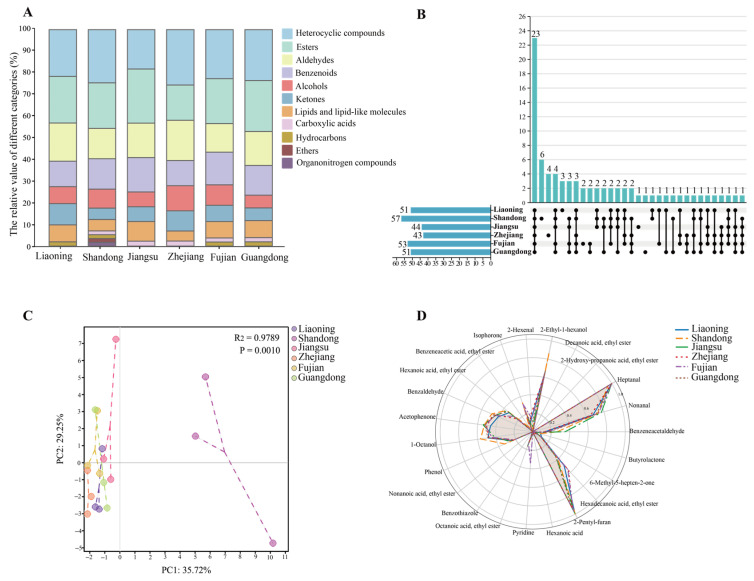
Volatile metabolite profiles of fresh *P. haitanensis* cultivated at different sites. (**A**) Histogram of aroma types. (**B**) Upset plot of odor-active compounds. (**C**) Principal component analysis (PCA) of laver samples based on the types and quantities of odor-active compounds. (**D**) Radar plot of odor-active compounds shared among samples from the six sea areas.

Among all detected volatile metabolites, a total of 78 compounds were identified as odor-active based on the FlavorDB2 database, which were classified into 10 chemical classes (Table S2). Esters constituted the largest group, with 18 compounds (Figure 4A), followed by heterocyclic compounds, benzenoids, and aldehydes. The number of OACs varied from 43 to 57 across cultivation regions (Figure 4B). The Shandong samples contained the greatest diversity, covering all 10 classes (57 compounds), with esters and heterocyclic compounds being most abundant. Fujian ranked second (53 compounds), whereas Zhejiang had the lowest number (43 compounds). In addition, the Zhejiang samples showed a relatively narrow source of odor compounds, dominated by heterocyclic compounds and aldehydes, and the proportion of alcohols was also the highest.

PCA based on the types and relative abundances of odor-active compounds revealed that, except for the samples from Liaoning which clustered separately, laver from the other regions shared relatively similar flavor profiles. This similarity was particularly evident among the southern sites of Zhejiang, Fujian, and Guangdong (Figure 4C).

In total, 23 flavor-active compounds were shared across all six cultivation regions (Figure 4B), including five esters and five heterocyclic compounds (Table S2). A radar plot constructed from the types and relative abundances of these 23 compounds (Figure 4D) showed that the overall flavor profiles of samples from different sites were highly similar, dominated by heptanal, 2-pentylfuran, nonanal, acetophenone, and 2-ethyl-1-hexanol. This indicates that the intrinsic flavor of *P. haitanensis* is consistent regardless of cultivation location. While the overall profile was similar, variations in the relative abundances of certain compounds may contribute to differences in flavor intensity among regions. Region-specific compounds were also detected: Shandong contained six unique volatiles, Zhejiang four, and fewer were found in the other regions. However, the ROAVs of these unique compounds were relatively low, indicating that they are unlikely to substantially influence the primary flavor characteristics of laver (Figure 4B, Table S2).

Table 2 lists the 13 OACs with ROAVs > 0.1, of which nine were shared across all six regions. Among these, four compounds had ROAVs > 1, including heptanal (citrus, fatty), 2-pentyl-furan (green beans, vegetable), 2-ethyl-1-hexanol (rose, green), and nonanal (citrus, orange peel), representing the core flavor constituents of *P. haitanensis*, consistent with the radar plot results. In the Shandong samples, three of these compounds were abundant, with relatively high levels of nonanal and 2-ethyl-1-hexanol. However, due to the low odor threshold of 2-pentyl-furan, its aroma dominated, with an ROAV of 100. In contrast, in the other regions, despite the higher relative abundance of nonanal, the sensory contribution was mainly from heptanal, which reached ROAVs of 100 in most regions and 79.56 in Zhejiang.

Regional differences were also evident in the distribution of dominant contributors. In the northern provinces (Liaoning, Shandong, and Jiangsu), the flavor profile was more diverse, with ≥6 compounds showing ROAV > 1. In the southern regions (Zhejiang, Fujian, and Guangdong), the flavor was mainly determined by four compounds: heptanal, 2-pentylfuran, 2-nonenal, and 2,6-nonadienal. Notably, 2-nonenal was absent in the Jiangsu and Liaoning samples but exhibited very high ROAVs in the others, especially in Zhejiang reaching 100. Similarly, 2,6-nonadienal was absent in Shandong and Jiangsu but contributed strongly in Liaoning, where its ROAV was comparatively high.

### 3.4. Environmental Parameters Across Cultivation Regions

Measurements of environmental parameters revealed clear regional differences (Table 3). For nitrogen availability, the Jiangsu site exhibited markedly higher concentrations of NH_4_^+^ and NO_2_^−^ than the other regions (3.12 ± 0.03 and 5.91 ± 0.14 μmol/L, respectively; *p* < 0.05), whereas Zhejiang was characterized by the highest NO_3_^−^ level (3.06 ± 0.17 μmol/L, *p* < 0.05). In contrast, both Guangdong and Shandong showed comparatively low overall nitrogen concentrations. Phosphate concentrations were generally higher in the southern provinces, with Zhejiang reaching the maximum value (1.43 ± 0.02 μmol/L). The levels in Zhejiang and Fujian were significantly greater than those in the northern sites of Shandong and Liaoning (*p* < 0.01). Correspondingly, the N/P ratio was highest in Jiangsu but lowest in Guangdong.

As for physicochemical parameters, salinity ranged from 25.50‰ to 31.40‰. Guangdong and Fujian both exceeded 30‰, whereas Zhejiang exhibited the lowest value. Electrical conductivity showed a similar pattern, being highest in Guangdong and lowest in Zhejiang. pH values differed only slightly among regions, remaining stable between 7.9 and 8.0. The deployment time of seedling nets varied among regions, with earlier placement in the north and later in the south. The cultivation period for the first harvest was 45 days across all sites. Then, seawater temperature at harvest ranged between 20.26 and 25.90 °C, and no clear trend of increasing temperature from north to south was observed.

Cultivation sites differed in their offshore distance. Farms in southern regions (Zhejiang, Fujian, and Guangdong) were situated closer to the coast, while those in Liaoning and Jiangsu were located further offshore. Sunshine duration was longer in the northern sites and shorter in the south, a pattern partly attributable to the earlier deployment of cultivation nets in the north.

### 3.5. Coefficient of Variation Analysis of Key Flavor Compounds and Environmental Parameters

Based on the screening criteria of TAV > 1 and ROAV > 0.1, key flavor compounds were identified, including 12 taste-active substances (e.g., Asp, Glu, Ala, and IMP) and 8 volatiles (e.g., heptanal, 2-pentylfuran, and 2-ethyl-1-hexanol). The CVs of these compounds and the environmental parameters across regions were calculated (Table 4). In general, a CV greater than 30% is considered indicative of relatively large variability.

Among the environmental parameters, nutrient concentrations showed the greatest variation. The CVs of NH_4_^+^, NO_3_^−^, and NO_2_^−^ all exceeded 100%, and the N/P ratio also fluctuated considerably (66.88%), whereas phosphate was comparatively stable (33.08%). By contrast, physicochemical parameters such as salinity, conductivity, temperature, total sunshine duration, and especially pH (0.62%) exhibited only minor variation among sites.

For taste-active amino acids and nucleotides, the degree of variability differed markedly among compounds. Glu, Asp, and Gly were relatively stable, with CVs below 20%, highlighting their role as core substances maintaining the fundamental taste. In contrast, Lys displayed the highest CV (122.51%), followed by Thr and Ile, indicating significant regional differences. OACs exhibited even greater regional divergence, far exceeding that of taste-active substances. 2-pentylfuran, 2-ethyl-1-hexanol, and hexadecanoic acid ethyl ester showed the highest variability, each with CVs above 150%, while heptanal and 1-octanol also displayed high variability.

### 3.6. Associations Between Environmental Factors and Flavor-Active Compounds

**Figure 5:**
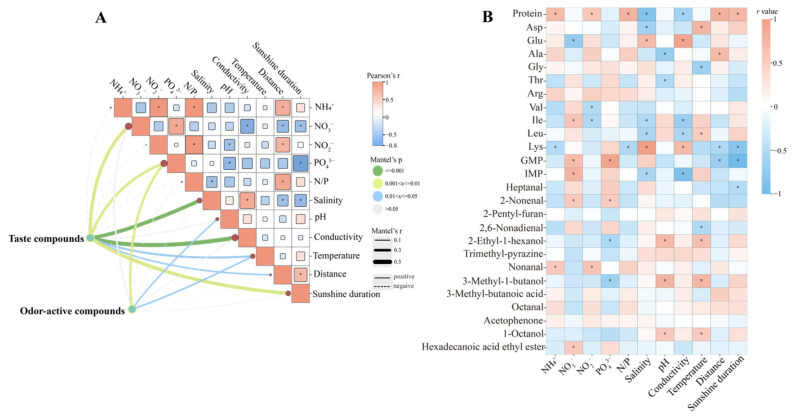
Correlations between environmental factors and taste-active compounds in *P. haitanensis*. (**A**) Mantel analysis of odor-active and taste-active compounds across different regions and their associations with environmental variables. (**B**) Heatmap showing correlations between environmental parameters and key flavor compounds. * indicates significant correlations (*p* < 0.05).

To clarify the influence of environmental parameters on flavor accumulation, Mantel tests were conducted between environmental factors and the matrices of key taste-active and odor-active compounds (Figure 5A). For taste-active substances, significant correlations were observed with several environmental variables. Among the nutrients, NO_3_^−^ and PO_4_^3−^ showed strong positive associations with the overall taste compound matrix (*r* > 0.3, *p* < 0.01). Physicochemical factors such as salinity and conductivity displayed strong positive correlations (*r* > 0.4, *p* < 0.001), and sunshine duration was likewise influential (*r* = 0.406, *p* < 0.01). For OACs, the overall profile was significantly correlated with PO_4_^3−^ (*r* = 0.269, *p* < 0.01), as well as with pH and temperature (*r* > 0.15, *p* < 0.05).

Pearson correlation analysis was further conducted to explore the relationships between environmental parameters and individual flavor-active compounds, with protein content also included in the analysis (Figure 5B). Protein content showed positive correlations with NH_4_^+^, NO_2_^−^, and the N/P ratio, as well as with sunshine duration (*p* < 0.05), but was negatively correlated with salinity and conductivity (*p* < 0.05). Several amino acids that contribute strongly to umami, including Asp, Glu, Ala, and Gly, tended to increase with NH_4_^+^, NO_2_^−^, and the N/P ratio, although these correlations were not statistically significant. By contrast, Glu was significantly negatively associated with NO_3_^−^ (*p* < 0.05). Bitter amino acids such as Lys showed significant positive associations with salinity and conductivity (*p* < 0.05). Notably, the nucleotides GMP and IMP exhibited significant positive correlations with both NO_3_^−^ and PO_4_^3−^ (*p* < 0.05). A few OACs exhibited significant positive correlations with nutrient concentrations. For instance, 2-nonenal was positively associated with NO_3_^−^ and PO_4_^3−^ (*p* < 0.05), while nonanal showed significant positive correlations with NH_4_^+^ and NO_2_^−^ (*p* < 0.05). Compared with nutrients, OACs appeared to be more strongly linked to physicochemical parameters, particularly pH and temperature. volatiles such as 3-methyl-1-butanol, and 1-octanol were all positively correlated with these factors (*p* < 0.05).

## 4. Discussion

Geographical relocation of agricultural species is well known to reshape product quality, as sensory traits are strongly influenced by environmental conditions [24]. A similar phenomenon is now emerging in marine aquaculture. *P. haitanensis* was historically cultivated in Zhejiang and Fujian, but its farming range has recently expanded northward to Liaoning, spanning nearly 15° of latitude, driven by climate warming and production demand [16]. Producers have noted alterations in texture, such as increased toughness in northern products. However, whether this geographic expansion also affects umami taste and aroma, the most critical determinants of sensory quality in *P. haitanensis*, remains unclear. Therefore, this study examined how cultivation across different regions influences flavor composition and evaluated the environmental factors associated with these variations. Particular attention was given to distinguishing intrinsic (genetically maintained) flavor components from those responsive to environmental modulation.

Although “ZHEDONG 1” was originally bred in Zhejiang, its growth performance showed no consistent geographic trend after transplantation. In some northern sites, blade length, width, and fresh weight even exceeded those in Zhejiang, suggesting that cross-regional cultivation did not impose substantial constraints on biomass production. In contrast, protein content remained relatively stable among regions, indicating that this trait is primarily under genetic control. The highest protein levels were still observed in Zhejiang. Correlation analysis showed positive associations between protein content and NH_4_^+^, NO_2_^−^, and the N/P ratio, consistent with reports that nitrogen availability promotes protein accumulation in laver and other macroalgae [25,26]. Although NO_3_^−^ enrichment has been shown to increase soluble protein [26], the lack of correlation in our study may reflect the use of total rather than soluble protein as an indicator. Sunshine duration was also positively correlated with protein content, potentially due to enhanced photosynthetic carbon assimilation [27]. By contrast, salinity was negatively correlated with protein levels, suggesting that under high-salinity conditions carbon and nitrogen flux may shift from structural protein synthesis toward osmolyte and stress-related metabolite production [28]. These results indicate that growth traits are largely genotype-driven, while nitrogen supply and osmotic conditions modulate resource allocation, laying the physiological basis for subsequent variation in taste- and aroma-related metabolites.

Nineteen taste-active amino acids and nucleotides were identified, among which Glu, Asp, Ala, IMP, and GMP exhibited high TAV due to their relatively high abundance and low sensory thresholds. These compounds, particularly Glu and IMP, constituted the primary basis of umami taste in *P. haitanensis*, consistent with previous findings [13]. Regional variation was evident: Guangdong samples showed the highest Glu levels, whereas Zhejiang samples contained the greatest IMP content. Despite the lower Glu concentration in Zhejiang, the strong sensory potency of IMP resulted in the highest EUC values at this site. The decline in EUC when cultivation shifted either northward or southward further indicates that IMP is the major determinant of umami intensity. Because IMP biosynthesis draws directly from nitrogen-assimilation pathways in purine metabolism, the elevated NO_3_^−^ concentration in Zhejiang seawater likely promoted IMP accumulation, supported by the positive correlation between NO_3_^-^ and IMP [26,29]. Coefficient of variation analysis reinforced this interpretation: Glu remained relatively stable across regions (CV < 20%), forming a core umami baseline, whereas IMP showed greater variability, reflecting its sensitivity to nitrogen supply conditions. Meanwhile, the regional enrichment of Lys, likely associated with osmotic adjustment under high salinity [30], contributed little to overall taste intensity but may subtly shift flavor balance. Together, these results suggest that umami taste in *P. haitanensis* consists of a stable amino acid-derived foundation, with IMP providing a key environmentally responsive component that governs regional sensory divergence.

Aroma is another key quality attribute of *P. haitanensis*, largely determined by odor-active volatile compounds [31]. In this study, more than 1100 volatile compounds were detected, yet only 78 exhibited odor activity, and 23 of these were consistently observed across all six cultivation regions. This relatively large conserved subset suggests that, although *P. haitanensis* synthesizes a diverse array of volatiles, only a fraction contributes directly to sensory perception, while many others likely function in physiological or ecological processes such as stress signaling and microbial interaction [32,33]. Zhejiang samples exhibited the greatest diversity of volatiles, including several region-specific compounds. Given that “ZHEDONG 1” was originally bred and domesticated under local environmental conditions [34], a broader volatile repertoire may reflect long-term adaptation to fluctuating coastal habitats. Importantly, the presence of a conserved set of odor-active compounds across all sites indicates a genetically anchored core aroma profile, while variation in the abundance of specific volatiles appears to underlie regional sensory differences.

Despite such variation, PCA and radar plot analyses showed that the overall flavor profiles of samples from different regions were largely similar. Among the 23 shared OACs, nine exhibited ROAVs above 0.1, indicating the presence of a stable core volatile set that defines the characteristic aroma of *P. haitanensis*. Radar plots combined with ROAVs further highlighted heptanal, 2-pentylfuran, nonanal, acetophenone, and 2-ethyl-1-hexanol as the principal contributors, which may indicate the presence of a genetically determined “core flavor” in *P. haitanensis* [35]. Nevertheless, regional cultivation introduced significant variability in the abundance of these core compounds, as reflected by high CV values for several OACs. For instance, heptanal, commonly produced from linoleic acid via lipoxygenase-mediated oxidation was the dominant contributor in most regions but was less prominent in Shandong [36]. In contrast, 2-pentylfuran, which imparts a distinct green bean note and is likewise associated with LOX-related lipid oxidation processes [37], reached an ROAV of 100 in the Shandong samples and became the primary contributor there. Previous studies have reported that *P. haitanensis* contains at least six LOX genes whose expression responds differently to environmental conditions [38], suggesting that environmental modulation of LOX-dependent fatty acid oxidation underlies the observed regional divergence in volatile composition. These results indicate that while core aroma identity is genetically maintained, the LOX pathway may serve as a key biochemical axis through which environmental factors modulate aromatic intensity and character.

The presence of region-specific OACs further differentiated the aroma profiles among cultivation sites. Shandong samples contained the greatest number of unique volatiles with ROAVs above 1, suggesting that northern conditions may favor a more complex aromatic composition, potentially reflecting stress-associated adjustments in metabolic allocation. In contrast, southern regions such as Zhejiang, Fujian, and Guangdong exhibited simpler profiles dominated by the conserved OAC set, thereby retaining the traditional sensory character of laver. Certain key odorants, such as 2-nonenal, which imparts a cucumber-like note [39], were abundant in Zhejiang but undetected in northern sites, underscoring the environmental dependence of specific volatile formation [40,41,42]. These regional differences involved not only the absolute abundance of individual compounds but also shifts in their relative proportions, which together shape the overall sensory perception of *P. haitanensis*.

Environmental correlations further supported these observations. Mantel and Pearson analyses revealed significant associations between OAC profiles and phosphate, temperature, and pH. These parameters have been reported to influence lipid oxidation and other secondary metabolic processes in algae and higher plants [12,43]. In our samples, key contributors with high ROAVs were primarily aldehydes (e.g., heptanal, nonanal, 2-nonenal). Phosphate availability has been linked to membrane lipid remodeling, which can indirectly affect aldehyde formation [44]. Likewise, seawater pH and temperature have been shown to influence both enzyme activity and the stability of volatile products [45]. 2-pentylfuran, commonly associated with fatty acid oxidation [46], was particularly abundant in Shandong samples, which may relate to the relatively higher water temperatures in this region. While the present study did not measure lipid-oxidation enzymes directly, the observed correlations suggest that environmental conditions likely influence the relative abundances of key aroma compounds rather than the presence or absence of the conserved volatile core, helping to explain regional differences in flavor intensity and nuance.

## 5. Conclusions

This study examined the flavor characteristics of *P. haitanensis* cultivated across six major regions in China using the same cultivar with a consistent genetic background. Both taste- and odor-active compounds varied with region. Glu, Asp, Ala, IMP, and GMP were identified as the principal contributors to umami taste, with IMP emerging as the key determinant of EUC, with nitrate availability strongly influencing IMP accumulation. A conserved set of volatiles, including heptanal, 2-pentylfuran, nonanal, acetophenone, and 2-ethyl-1-hexanol was consistently detected across all sites, forming the core aroma profile of *P. haitanensis*. However, the relative abundances of these volatiles varied significantly with region, and additional site-specific compounds contributed to distinct sensory nuances. These findings demonstrate that *P. haitanensis* maintains a genetically determined core flavor, but environmental conditions modulate its intensity and complexity, providing a basis for improving site selection and ensuring stable product quality.

## Figures and Tables

**Table 1:** Taste-active amino acids and nucleotides of *P. haitanensis* from different cultivation regions and their corresponding taste activity values (TAVs).

Amino Acids	Taste Attribute	Threshold (mg g^−1^)	Liaoning		Shandong		Jiangsu		Zhejiang		Fujian		Guangdong	
			Content (mg g^−1^ DW)	TAV	Content (mg g^−1^ DW)	TAV	Content (mg g^−1^ DW)	TAV	Content (mg g^−1^ DW)	TAV	Content (mg g^−1^ DW)	TAV	Content (mg g^−1^ DW)	TAV
Asp	Umami (+)	1	3.72 ± 0.33 ^b^	3.72	3.75 ± 0.08 ^b^	3.75	3.48 ± 0.15 ^b^	3.48	3.93 ± 0.66 ^b^	3.93	2.59 ± 0.34 ^a^	2.59	3.38 ± 0.11 ^ab^	3.38
Glu	Umami (+)	0.3	16.34 ± 0.09 ^b^	54.47	14.71 ± 0.25 ^b^	49.03	16.31 ± 0.25 ^b^	54.37	11.37 ± 1.11 ^a^	37.90	15.61 ± 0.36 ^b^	52.03	18.84 ± 1.63 ^c^	62.80
Ala	Sweet (+)	0.6	67.64 ± 0.61 ^c^	112.73	24.08 ± 1.51 ^a^	40.13	60.54 ± 3.55 ^a^	100.90	47.39 ± 1.95 ^b^	78.98	26.42 ± 0.50 ^a^	40.03	60.96 ± 6.74 ^c^	101.60
Gly	Sweet (+)	1.3	1.98 ± 0.05	1.52	1.63 ± 0.19	1.25	2.03 ± 0.41	1.56	1.77 ± 0.12	1.36	2.24 ± 0.52	1.72	2.01 ± 0.22	1.55
Thr	Sweet (+)	2.6	3.59 ± 0.11 ^b^	1.38	1.21 ± 0.21 ^a^	0.47	1.80 ± 0.04 ^a^	0.69	3.95 ± 0.46 ^b^	1.52	2.03 ± 0.21 ^a^	0.78	5.18 ± 0.76 ^c^	1.99
Arg	Bitter/Sweet (+)	0.5	0.22 ± 0.1 ^a^	0.44	0.84 ± 0.03 ^b^	1.68	1.14 ± 0.13 ^c^	2.28	0.73 ± 0.06 ^b^	1.46	0.90 ± 0.14 ^bc^	1.80	0.74 ± 0.14 ^b^	1.48
Val	Sweet/Bitter (−)	0.4	1.52 ± 0.11 ^c^	3.80	0.87 ± 0.05 ^ab^	2.18	0.70 ± 0.10 ^a^	1.75	1.43 ± 0.19 ^c^	3.58	0.95 ± 0.02 ^ab^	2.38	1.08 ± 0.20 ^b^	2.70
Met	Bitter/Sweet (−)	0.3	0.20 ± 0.01 ^b^	0.67	0.09 ± 0.02 ^a^	0.30	0.08 ± 0.01 ^a^	0.27	0.09 ± 0.03 ^a^	0.30	0.11 ± 0.01 ^a^	0.37	0.19 ± 0.01 ^b^	0.63
Ile	Bitter (−)	0.9	1.27 ± 0.04 ^b^	1.41	2.11 ± 0.23 ^c^	2.34	0.75 ± 0.07 ^a^	0.83	2.53 ± 0.43 ^c^	2.81	1.34 ± 0.07 ^b^	1.49	0.46 ± 0.07 ^a^	0.48
Phe	Bitter (−)	0.9	0.35 ± 0.01 ^a^	0.39	0.79 ± 0.12 ^b^	0.88	0.67 ± 0.10 ^b^	0.74	0.76 ± 0.14 ^b^	0.84	0.22 ± 0.03 ^a^	0.24	0.65 ± 0.16 ^b^	0.72
Leu	Bitter (−)	1.9	11.91 ± 0.15 ^ab^	6.27	16.31 ± 0.36 ^b^	8.58	10.63 ± 4.54 ^ab^	5.59	16.04 ± 4.88 ^b^	8.44	10.11 ± 0.26 ^ab^	5.32	6.47 ± 0.76 ^a^	3.41
His	Bitter (−)	0.2	0.04 ± 0.01	0.20	0.05 ± 0.04	0.25	0.03 ± 0.01	0.15	0.05 ± 0.03	0.25	0.04 ± 0.01	0.20	0.03 ± 0.01	0.15
Lys	Bitter (−)	0.5	0.56 ± 0.03 ^a^	1.12	0.49 ± 0.15 ^a^	0.98	0.29 ± 0.05 ^a^	0.58	0.75 ± 0.25 ^a^	1.50	7.57 ± 0.30 ^b^	15.14	10.62 ± 0.16 ^c^	21.24
Ser	Sweet (+)	1.5	0.76 ± 0.09	0.51	0.56 ± 0.12	0.37	0.70 ± 0.17	0.47	1.01 ± 0.21	0.67	0.70 ± 0.23	0.47	0.90 ± 0.28	0.60
Total			110.09 ± 0.76 ^c^		67.50 ± 1.17 ^a^		99.16 ± 0.56 ^b^		91.80 ± 3.57 ^b^		70.82 ± 2.07 ^a^		111.51 ± 6.12 ^c^	
AMP	Umami (+)	0.5	10.52 ± 1.24 ^a^	0.21	10.86 ± 0.83 ^a^	0.22	13.47 ± 0.81 ^ab^	0.27	18.46 ± 1.20 ^c^	0.37	14.93 ± 1.51 ^b^	0.30	14.89 ± 2.08 ^b^	0.30
GMP	Umami (+)	0.125	8.03 ± 1.34 ^a^	0.64	14.1 ± 0.52 ^b^	1.13	19.92 ± 1.30 ^c^	1.59	25.62 ± 1.84 ^d^	2.05	27.11 ± 3.17 ^d^	2.17	26.74 ± 1.02 ^d^	2.14
IMP	Umami (+)	0.25	70.07 ± 4.15 ^a^	2.80	147.83 ± 2.49 ^d^	5.91	129.27 ± 8.95 ^c^	5.17	205.37 ± 1.05 ^e^	8.21	109.47 ± 6.90 ^b^	4.38	71.92 ± 8.77 ^a^	2.88
CMP	Tasteless	0.8	15.56 ± 2.03 ^cd^	0.19	11.71 ± 2.19 ^bc^	0.15	18.2 ± 1.02 ^d^	0.23	14.61 ± 1.82 ^cd^	0.18	7.17 ± 1.25 ^a^	0.09	9.01 ± 0.68 ^ab^	0.11
UMP	Tasteless	0.4	10.56 ± 1.25 ^b^	0.26	17.03 ± 0.14 ^cd^	0.43	15.90 ± 1.7 ^cd^	0.40	18.7 ± 0.26 ^d^	0.47	15.24 ± 1.36 ^c^	0.38	4.25 ± 0.76 ^a^	0.11
Total	Total		114.75 ± 3.44 ^a^		201.53 ± 1.06 ^d^		196.77 ± 6.62 ^d^		282.76 ± 2.71 ^e^		173.93 ± 6.63 ^c^		126.81 ± 7.32 ^b^	

TAV is obtained by dividing the concentration of the metabolite by its taste threshold. ‘+’ denotes pleasant; ‘−’ denotes unpleasant; AA represents amino acid. The different superscript letters in the same row indicate significant difference (*p* < 0.05).

**Table 2:** Relative abundance and ROAVs of odor-active compounds in *P. haitanensis* from different cultivation regions.

Substance	Odor Character	Taste Threshold	Liaoning		Shandong		Jiangsu		Zhejiang		Fujian		Guangdong	
			Content (%) *	ROAV	Content (%)	ROAV	Content (%)	ROAV	Content (%)	ROAV	Content (%)	ROAV	Content (%)	ROAV
Heptanal	Citrus, Fatty	0.003	0.57 ± 0.44	100	0.43 ± 0.11	55.39	0.49 ± 0.38	100	1.61 ± 0.84	79.56	2.28 ± 0.86	100	1.78 ± 2.30	100
2-Nonenal	Fatty, Cucumber	0.0002	/	/	0.02 ± 0.03	38.55	/	/	0.14 ± 0.12	100	0.06 ± 0.09	41.81	0.09 ± 0.06	77.43
2-Pentylfuran	Green Beans, Vegetable	0.006	0.55 ± 0.07	48.11	1.56 ± 2.20	100	0.53 ± 0.42	54.4	0.28 ± 0.20	6.96	0.37 ± 0.19	8	0.25 ± 0.28	6.98
2,6-Nonadienal	Cucumber, Green	0.02	0.17 ± 0.09	4.56	/	/	/	/	0.18 ± 0.08	1.31	0.27 ± 0.10	1.78	0.12 ± 0.17	1.01
2-Ethyl-1-hexanol	Rose, Green	0.198	0.41 ± 0.19	1.09	4.68 ± 2.19	9.12	0.38 ± 0.33	1.17	0.29 ± 0.22	0.22	0.38 ± 0.39	0.25	0.49 ± 0.12	0.42
Trimethyl-pyrazine	Roasted Nuts, Cocoa, Peanuts	0.023	0.05 ± 0.02	1.11	0.26 ± 0.10	4.4	0.09 ± 0.13	2.4	/	/	0.08 ± 0.06	0.47	0.14 ± 0.16	1.02
Nonanal	Aldehyde, Citrus	1	2.46 ± 0.96	1.28	4.49 ± 1.49	1.73	7.14 ± 3.18	4.37	1.72 ± 0.75	0.25	4.86 ± 1.35	0.64	3.66 ± 1.81	0.62
3-Methyl-1-butanol	Sweet, Malty	1.69	/	/	6.13 ± 0.33	1.4	/	/	/	/	/	/	/	/
3-Methyl-butanoic acid	Rancid Cheese, Sweaty	0.02	0.16 ± 0.14	4.08	/	/	0.06 ± 0.03	1.93	0.01 ± 0.01	0.1	0.05 ± 0.03	0.32	0.06 ± 0.05	0.52
Octanal	Lemon, Citrus	2.5	0.74 ± 1.05	0.15	1.24 ± 0.96	0.19	1.28 ± 1.81	0.31	/	/	/	/	0.52 ± 0.73	0.03
Acetophenone	Sweet, Almond	0.24	0.05 ± 0.04	0.1	0.10 ± 0.04	0.17	0.12 ± 0.10	0.31	0.08 ± 0.05	0.05	0.09 ± 0.06	0.05	0.10 ± 0.04	0.07
1-Octanol	Penetrating	0.9	0.25 ± 0.20	0.14	0.63 ± 0.19	0.27	0.11 ± 0.15	0.07	0.22 ± 0.20	0.04	0.15 ± 0.21	0.02	0.25 ± 0.19	0.05
Hexadecanoic acid, ethyl ester	Wax	2	0.61 ± 0.31	0.16	0.20 ± 0.12	0.04	0.14 ± 0.05	0.04	1.41 ± 1.90	0.1	1.20 ± 0.89	0.08	0.13 ± 0.08	0.01

* Relative abundance of normalized odor activity compound.

**Table 3:** Environmental parameters in different cultivation regions.

Parameters	Liaoning (7.19–9.01) *	Shandong (8.02–9.15)	Jiangsu (8.17–9.30)	Zhejiang (9.02–10.16)	Fujian (9.17–10.31)	Guangdong (10.13–11.06)
NH_4_^+^ (μmol L^−1^)	0.95 ± 0.06 ^c^	0.59 ± 0.02 ^b^	3.12 ± 0.03 ^d^	0.22 ± 0.01 ^a^	0.24 ± 0.01 ^a^	0.20 ± 0.02 ^a^
NO_3_^−^ (μmol L^−1^)	0.13 ± 0.01 ^a^	0.23 ± 0.04 ^a^	0.34 ± 0.03 ^a^	3.06 ± 0.17 ^d^	1.56 ± 0.13 ^c^	0.66 ± 0.03 ^b^
NO_2_^−^ (μmol L^−1^)	1.63 ± 0.03 ^c^	0.49 ± 0.06 ^a^	5.91 ± 0.14 ^d^	0.39 ± 0.05 ^a^	1.06 ± 0.07 ^b^	1.06 ± 0.09 ^b^
PO_4_^3−^ (μmol L^−1^)	0.61 ± 0.04 ^a^	0.53 ± 0.01 ^a^	1.03 ± 0.01 ^b^	1.43 ± 0.02 ^d^	1.26 ± 0.07 ^c^	1.09 ± 0.04 ^b^
N/P	4.45 ± 0.23 ^c^	2.48 ± 0.09 ^b^	9.08 ± 0.15 ^d^	2.56 ± 0.11 ^b^	2.27 ± 0.13 ^b^	1.77 ± 0.15 ^a^
Salinity (‰)	26.60 ± 0.01 ^b^	28.50 ± 0.01 ^d^	26.70 ± 0.01 ^c^	25.50 ± 0.01 ^a^	30.60 ± 0.01 ^e^	31.40 ± 0.01 ^f^
pH	7.95 ± 0.01 ^b^	8.01 ± 0.02 ^c^	7.88 ± 0.01 ^a^	7.89 ± 0.02 ^a^	7.97 ± 0.01 ^b^	7.90 ± 0.01 ^a^
Conductivity (S m^−1^)	46.57 ± 0.12 ^c^	46.90 ± 0.01 ^d^	43.47 ± 0.25 ^b^	38.10 ± 0.26 ^a^	46.37 ± 0.06 ^c^	51.13 ± 0.06 ^e^
Temperature (°C)	22.79 ± 0.05 ^b^	25.90 ± 0.06 ^e^	22.92 ± 0.37 ^b^	24.33 ± 0.45 ^d^	20.26 ± 0.05 ^a^	23.47 ± 0.03 ^c^
Distance (m)	8520.01 ± 10.01 ^a^	1452.00 ± 10.58 ^d^	8256.00 ± 31.48 ^e^	653.00 ± 14.11 ^a^	777.33 ± 15.37 ^b^	846.00 ± 46.36 ^c^
Sunshine time (h)	623.77	597.37	569.73	544.45	527.01	514.39

* indicates the cultivation duration for *P. haitanensis* at each site. Distance refers to the shortest distance between the cultivation area and the shoreline. The different superscript letters in the same row indicate significant difference (*p* < 0.05).

**Table 4:** Coefficient of variation in environmental parameters and key flavor compounds across different regions.

Environment	%	Components	%
NH_4_^+^	116.52	Asp	14.84
NO_3_^−^	104.54	Glu	15.09
NO_2_^−^	108.51	Ala	36.13
PO_4_^3−^	33.08	Gly	16.16
N/P	66.88	Thr	48.12
Salinity	7.68	Arg	38.57
pH	0.62	Val	28.56
Conductivity	8.74	Ile	52.25
Temperature	7.39	Leu	34.47
Distance	103.14	Lys	122.51
Total sunshine duration	6.85	GMP	35.92
		IMP	38.32
		Heptanal	109.37
		2-Pentyl-furan	174.73
		2-Ethyl-1-hexanol	167.35
		Nonanal	61.60
		Acetophenone	70.72
		1-Octanol	95.55
		Hexadecanoic acid, ethyl ester	164.96
		Benzaldehyde	44.09

## Data Availability

The original contributions presented in this study are included in the article/Supplementary Material. Further inquiries can be directed to the corresponding author.

## Supplementary Materials

The following supporting information can be downloaded at https://www.mdpi.com/article/10.3390/foods15010181/s1, Table S1: Volatile compounds of *Pyropia haitanensis* across different cultivation sites. Table S2: Odor activity compounds of *Pyropia haitanensis* across different cultivation sites. Figure S1: The upset diagram of volatile compounds across different cultivation sites. Figure S2: Representative Total Ion Chromatograms (TIC) for *P. haitanensis* samples from the six different cultivation regions.

## Abbreviations

The following abbreviations are used in this manuscript:

CV	coefficient of variation
EUC	equivalent umami concentration
FAA	free amino acid
GC×GC–TOFMS	comprehensive two-dimensional gas chromatography–time-of-flight mass spectrometry
LC-MS	liquid chromatography–mass spectrometry
LOX	lipoxygenase
MSG	monosodium glutamate
OAC	odor-active compound
PCA	principal component analysis
ROAV	relative odor activity value
TAV	taste activity values
