# Supplementary figures and images for: Influence of Cross-Regional Cultivation on the Flavor Characteristics of Pyropia haitanensis
Source: Foods. 2026 Jan 5;15(1):181. doi: 10.3390/foods15010181 (PMC12785560; doi:10.3390/foods15010181)

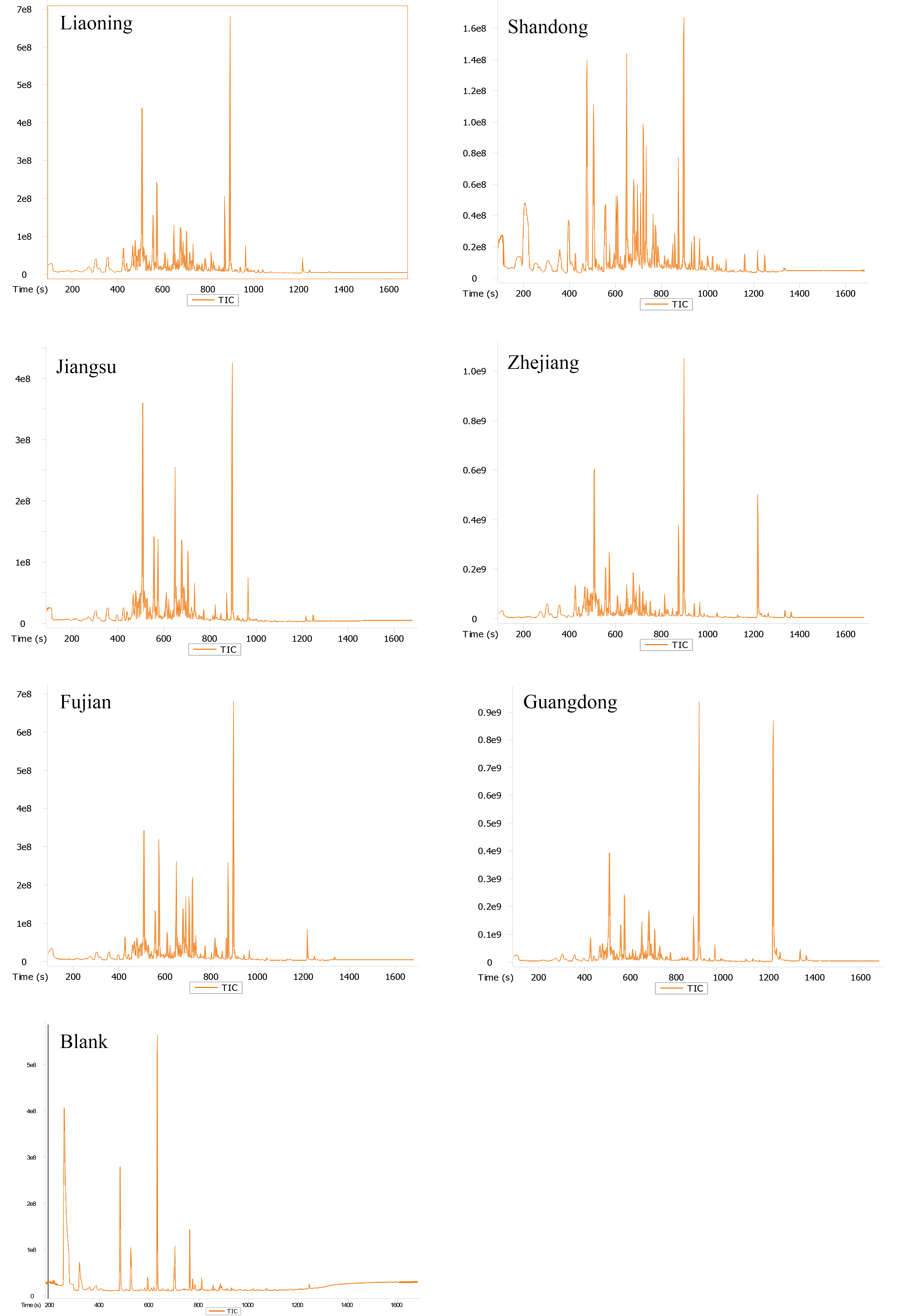

Supplement: Supplementary file 1 [file foods-15-00181-s001.zip › Fig. S2.png]

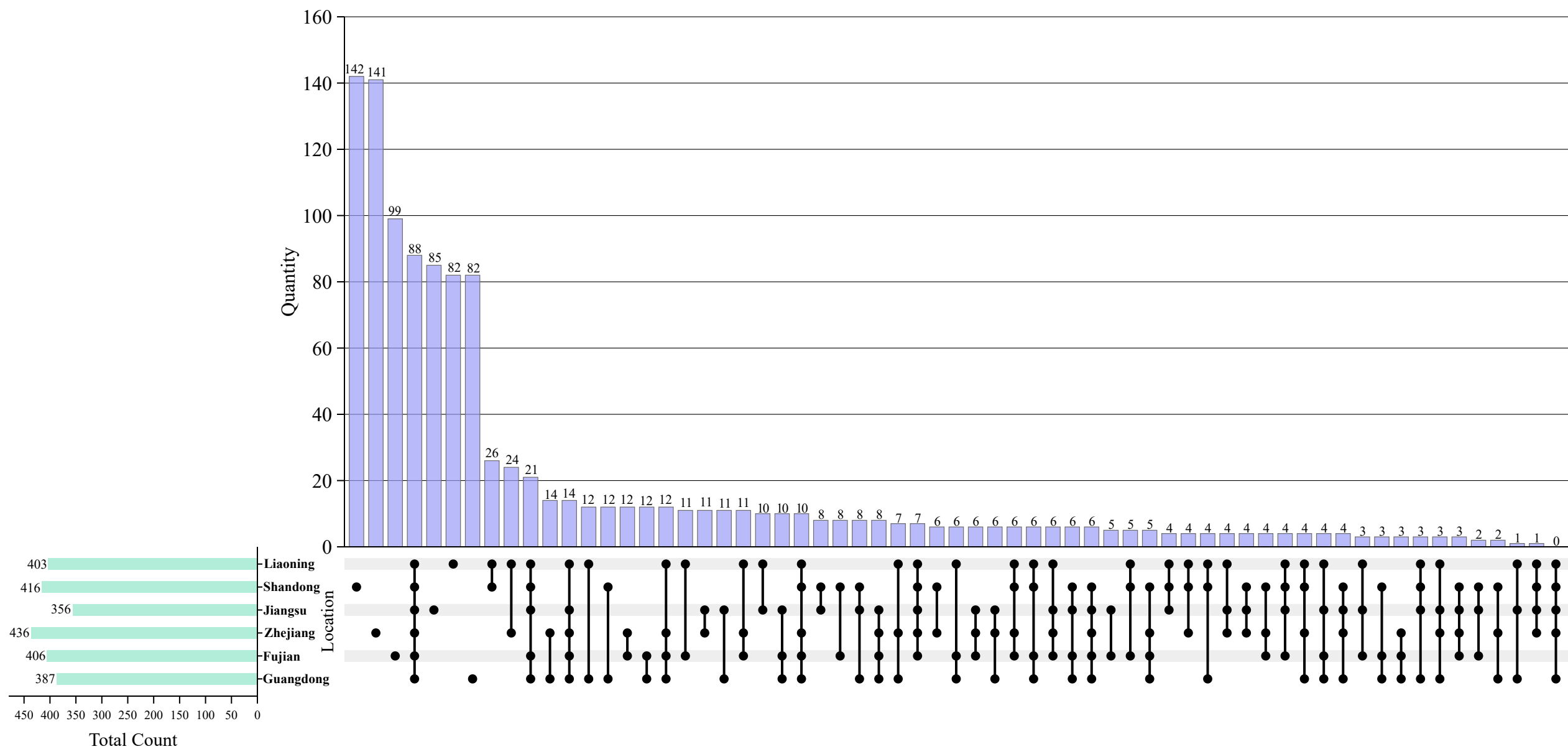

Supplement: Supplementary file 1 [file foods-15-00181-s001.zip › Fig. S1.pdf]
